# Supplementary material for: SyNDI: synchronous network data integration framework
Source: BMC Bioinformatics. 2018 Nov 6;19:403. doi: 10.1186/s12859-018-2426-5 (PMC6219086; doi:10.1186/s12859-018-2426-5)
Supplement: Supplementary file 5 — Files of the example presented in Additional file 4. This additional file is a zip package that contains all files used in Additional file 4. Detailed descriptions of each of these files are presented in a README file included in this zip package. (GZ 30411 kb) [file 12859_2018_2426_MOESM5_ESM.gz › SyNDITools/Meme2Fimo/meme_4.9.0_4.tar.gz!/meme_4.9.0/website/html/help_psp.html]

MEME Suite - Position-Specific Priors


|  |  |
| --- | --- |
|  | MEME Suite The MEME Suite web application requires the use of JavaScript   Javascript doesn't seem to be available on your browser.  - **Discriminative Motif Discovery**    An approach to motif discovery where you are attempting   to find a motif that is not only specific to sequences   of interest, but is *not* specific to other similar   sequences.        An example is with finding a DNA   transcription factor binding site, where you may have   some sequences that are likely to contain binding sites,   and other sequences that are unlikely to contain binding   sites (perhaps all found in one experiment). In this   case, you want to use the likely   (‘positive’) sequences to search for the   motif, while using the unlikely (‘negative’)   sequences to guide the search away from motifs that are   not only specific to the positive but also the negative   sequences. The approach we use here is to calculate a   position-specific prior that we use   as an additional input to MEME, to inform the search as to the   probability of a motif starting at any given starting   point in the positive sequences. The approach we use is   based on the *discriminative prior* devised by   Narlikar *et al*. We modify their approach to   search for the "best" initial motif width, and to handle   protein sequences, using *spaced   triples*.        The principle behind a discriminative   prior arises from the question: What fraction of all occurrences of the *w*-mer   (subsequence *w* long) at that site are found in the   positive set? We score each position by counting   how often the *w*-mer starting there occurs in the   positive set (call this count #**X**) and how often   it occurs in the negative set (this count is   #**Y**). The score for the location is then:   #**X**       If we find any ambiguous letters in a *w*-mer   (e.g. `N`, which stands for any base in   DNA), we count any such *w*-mer as if it occurs once.        For protein sequences, instead of requiring an exact   match of a *w*-wide subsequence, we break the   subsequence down into *spaced triples*, in which we   only care about the first and last letter, and one of   the interior letters. See below for a more detained   description of spaced triples.     The actual formula we calculate includes pseudocounts so   that small numbers of occurrences in the positive set without   any occurrences in the negative set do not cause false   peaks in the PSP. Once we have calculated scores, we   scale them to lie between 0.1 and 0.9 inclusive. We   convert the scaled scores to probabilities to complete   the calculation of the PSP. As with DNA sequences, we   treat any triple containing an ambiguous symbol as if it   only occurs once.     More detail follows.     ---    - **Position-Specific Priors**   - **Negative Set**   - **Positive Set**   - **Spaced Triples**    ---    This paper contains results illustrating the benefits of PSPs:     Timothy L. Bailey, Mikael Bodén, Tom Whitington, and   Philip Machanick, "The value of   position-speciﬁc priors in motif discovery using   MEME",   *BMC Bioinformatics*, 11(1):179, 2010.   [full text] |
